# Surgical management of hypertrophic cardiomyopathy

**DOI:** 10.1007/s12055-026-02180-1

**Published:** 2026-01-26

**Authors:** Chandrasekaran Ananthanarayanan, SriKrishna Reddy Modugula, Vincent Chauvette, Daniel Ragheb, Rohun Bhagat, Nicholas Smedira

**Affiliations:** Department of Thoracic and Cardiovascular Surgery, Heart Vascular and Thoracic Institute, Cleveland Clinic, 9500 Euclid Avenue, Cleveland, OH 44195 USA

**Keywords:** Alcohol septal ablation, Hypertrophic cardiomyopathy, Septal Myectomy

## Abstract

**Background:**

Hypertrophic cardiomyopathy (HCM) is a dynamic disease with a spectrum of clinical presentations ranging from incidental diagnosis to sudden cardiac death. Symptomatic patients are initially managed medically, and newer drugs are currently under investigation. Invasive therapy is needed for patients with intractable symptoms despite maximal medical therapy, and surgical myectomy is the gold standard treatment with better long-term survival.

**Results:**

Over three decades, more than 4000 patients underwent surgical myectomy at the Cleveland Clinic. We conducted a detailed analysis of patients who underwent surgery for HCM between 2005 and 2015. Within this study period, 1549 patients underwent surgical myectomy. Their mean pre-operative peak left ventricular outflow tract (LVOT) gradient was 63 ± 4.6 mmHg, and it reduced to 15 ± 8.9 mmHg after surgery. Mean aortic cross-clamp time was 28 ± 10 min for isolated septal myectomy, and the mean mass of muscle resected was 8.1 ± 3.7 g. Complications include new incidences of pacemaker insertion in 4.2% of the patients, iatrogenic ventricular septal defects in two patients, and the overall operative mortality was 0.38%. Mean hospital stay was 6 days, and the majority of the patients are in New York Heart Association (NYHA) class I in their post-operative follow-up.

**Conclusion:**

Septal myectomy is a curative therapy for most patients of HCM who are symptomatic despite maximal medical management. Clear understanding of individual septal anatomy and mechanisms of LVOT obstruction are key to successful surgical outcomes. Septal myectomy can be done safely with excellent long-term results.

## Introduction

Hypertrophic cardiomyopathy (HCM) is a primary disease of the myocardium known from the early days of cardiac surgery [1]. Over the years, our understanding of the disease has improved, and we now know that HCM has a spectrum of presentations and anatomical varieties such as hypertrophic obstructive, non-hypertrophic obstructive, and hypertrophic non-obstructive cardiomyopathy, all grouped under the roof of HCM [2, 3]. Even now, HCM remains a tough fortress to conquer, with limited centers of excellence which can consistently provide better long-term outcomes in managing patients with HCM [4]. In this article, we review the anatomy, pre-operative planning, surgical techniques, and outcomes associated with the surgical management of HCM.

## Historical perspectives

Even though pathological hypertrophy of the interventricular septum was identified as early as 1868, it was not until 1957 that Brock made one of the earliest descriptions of HCM after noticing an abnormal fibrous thickening in the septal endocardium of autopsied hearts but puzzled by the absence of valvular aortic stenosis [5]. The characteristic contact lesion he identified is now understood to be caused by the septo-mitral contact and is one of the hallmark features of classical HCM. Soon after this, Teare noticed “asymmetric septal hypertrophy” in young people who had sudden cardiac death [6].

### Evolution of septal myectomy

Early attempts at surgical correction were largely unsuccessful due to a poor understanding of the disease process, resulting in persistent gradients even after open-heart surgery [7]. The first successful surgical myectomy was done by Cleland in London in 1958 [1]. With very minimal resection of the bulging septal muscle, the patient had great symptom relief. Dr. Cleland did similar operations in a few other patients and presented his results in 1963 [8].

The first systematic attempts of surgery for HCM started at the Mayo Clinic by Dr. Kirklin in the 1950s and later developed by Dr. Frye [9]. Dr. Andrew Glenn Morrow at National Institutes of Health (NIH) Bethesda hypothesized that obstruction in HCM was because of an anatomical sphincter at the sub-valvular level. He believed splitting open the sphincter would correct the problem, an adaptation of the surgical principle behind Heller’s myotomy [10]. He devised the famous “Morrow operation” which involves two separate vertical incisions in the septum which was connected by a transverse myotomy resecting a portion of the hypertrophied septum. Soon, the Morrow operation became popular in other parts of North America and underwent a series of modifications to become what is now the gold standard treatment of HCM. Though the operation he pioneered has saved thousands of lives, it is a less well-known fact that Dr. Morrow was diagnosed with HCM by Dr. Eugene Braunwald, his good friend and a renowned cardiologist. Dr. Morrow refused to undergo surgery and died of complications due to the disease. The case of Dr. Morrow illustrates important aspects of HCM. All three of his children had HCM (autosomal dominant inheritance), and two of them had aggressive disease (variable penetrance). His daughter suffered from the non-hypertrophic sub-type and underwent heart transplantation, while his son had obstructive disease and was treated successfully with septal myectomy, the procedure devised by his own father [11].

The Morrow operation of the 1960s had more emphasis on depth rather than length of excision [12]. In the 1990 s, Dr. Bruno J. Messmer from Germany identified the inherent disadvantage of the Morrow operation (incomplete distal resection) and developed the concept of extended septal myectomy [13]. He stressed the importance of resection beyond the basal septum towards the apex for adequate relief of obstruction. The technique he described involves additional resection of the lateral and posterior margins of the septum, and trimming of the papillary muscles and freeing up its abnormal attachments, followed by a detailed inspection of the mitral valve [14].

Dr. Daniel G. Swistel from New York University introduced the resection, plication, and release (RPR) operation, which involves septal resection, plication of elongated anterior mitral leaflet (AML), and release of abnormal muscular and chordal attachments [15]. In 1999, Sir Magdy Yacoub from the Royal Brompton & Harefield Hospitals postulated that excessive deposition of fibrous tissue in between right and left fibrous trigones is an important pathological change in HCM which alters the hinge mechanism, pulling the AML anteriorly and eventually reducing the size of left ventricular outflow tract (LVOT). His technique involves mobilization of right and left fibrous trigones, restoring the anatomy of the LVOT [16].

The Cleveland Clinic experience with surgery for HCM began in the 1970s. Over the last five decades, we have incorporated techniques from Dr. Yacoub and others, and standardized our surgical strategies into a three-step resection procedure, which is described later in this article.

### Definition

HCM is defined as ventricular hypertrophy more than 15 mm, a ratio of thickness between the septal and posterior wall of more than 1.3, in the absence of secondary causes such as valvular disease, hypertension, or systemic pathologies [1, 2]. The cutoff of wall thickness is reduced to 13 mm in patients with known family history [17].

### Sub-types

HCM patients can be broadly classified into three anatomical sub-types. It should be kept in mind that they are not mutually exclusive; rather, they form a spectrum of anomalies with significant overlap between the sub-types.

**Fig. 1:**
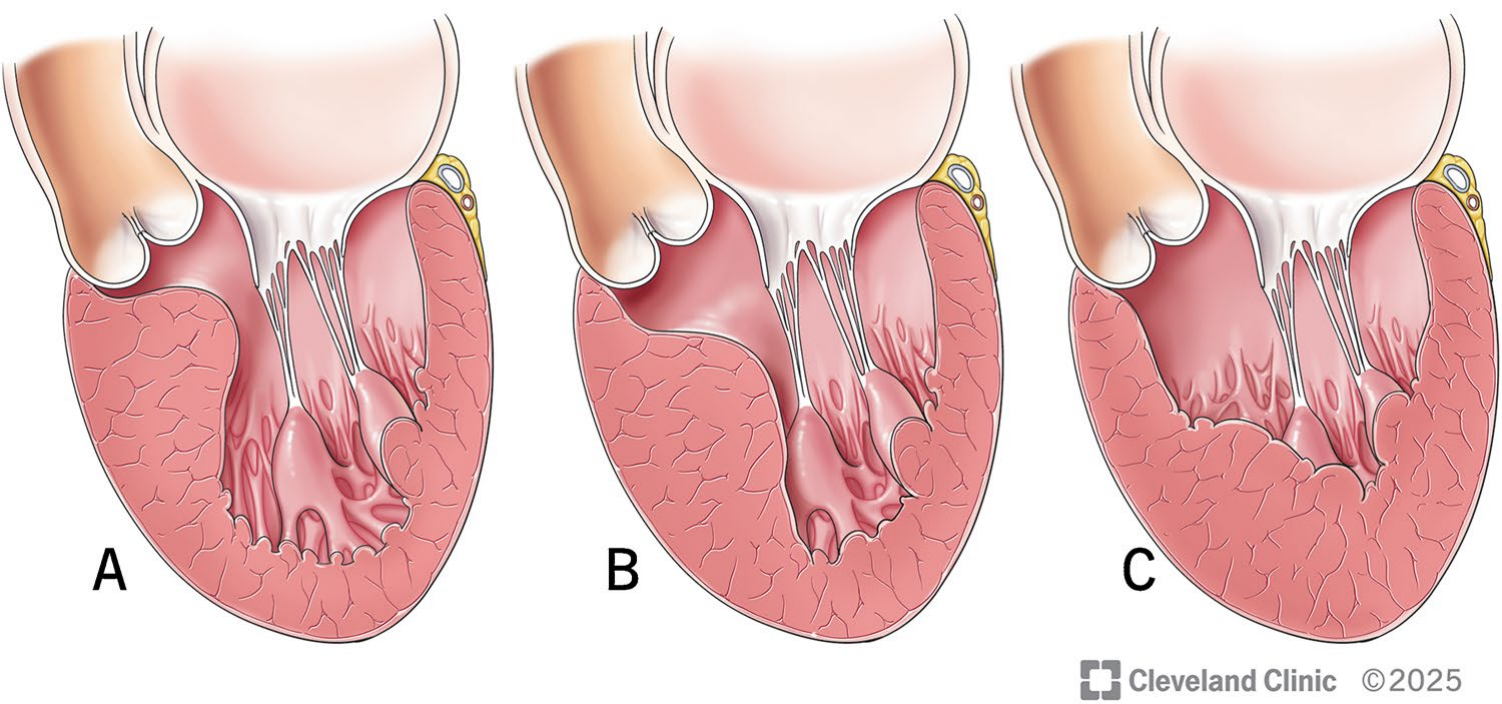
Types of hypertrophic cardiomyopathy. **A**-Subaortic or Basal, **B**-MidVentricular, **C**-Apical

Subaortic or basal HCM: Predominant hypertrophy of the basal interventricular septum (IVS). This variety is commonly associated with LVOT obstruction and systolic anterior motion (SAM) of the mitral valve [18] (Fig. 1A).

Mid-ventricular HCM: This commonly co-exists with either subaortic variety or apical variety. Exclusive mid-ventricular HCM occurs in less than 10% of the patients. Mid-ventricular obstruction can also cause SAM [19] (Fig. 1B).

Apical HCM: Though historically considered benign, recent data suggests apical HCM poses potentially serious risks including a higher risk of arrhythmias, heart failure, and sudden cardiac death and need for implantable cardioverter defibrillator (ICD) placement and heart transplantation as a last resort [20] (Fig. 1C).

## Patho-anatomy of LVOTO

**Fig. 2:**
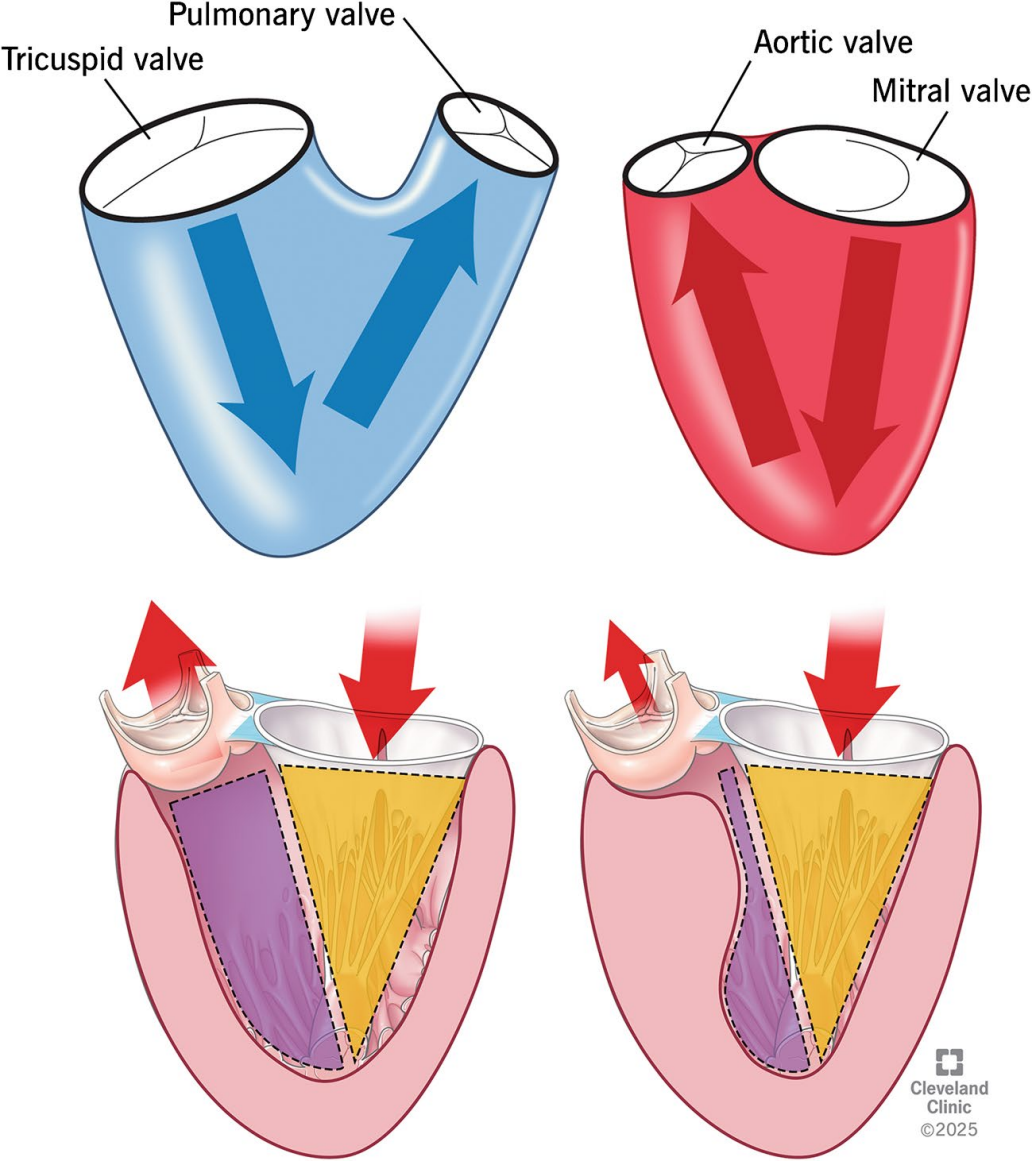
Difference in inflow and outflow tracts of right ventricular (RV) versus left ventricular (LV), LV outflow tract in normal versus hypertrophic cardiomyopathy (HCM) heart

**Fig. 3:**
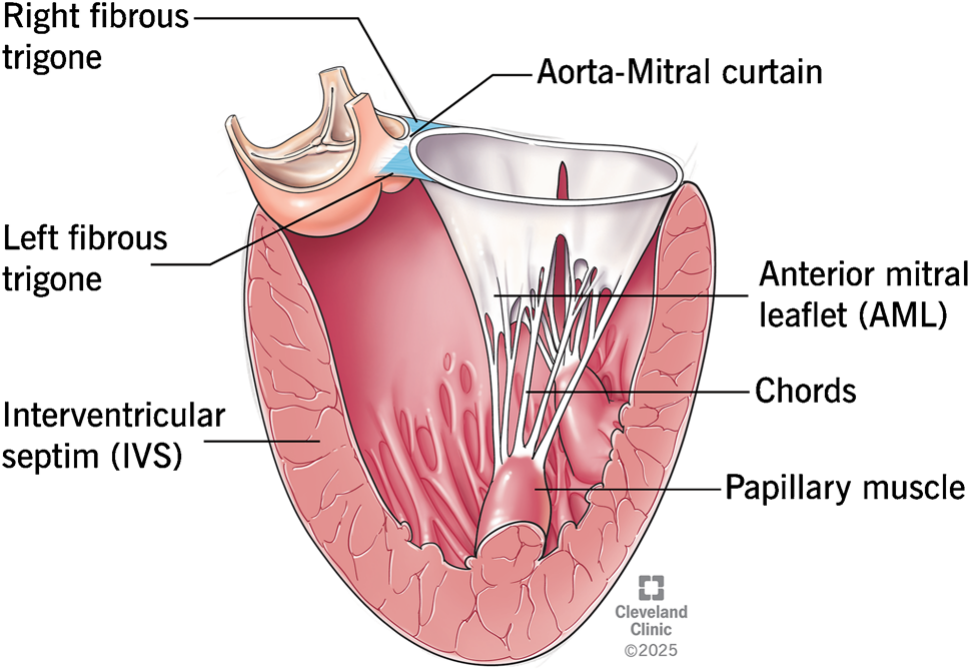
Structures of the heart contributing to left ventricular outflow tract

Interventricular septum (IVS) of a normal heart is not vertically oriented but is instead placed at an angle of 45° relative to the sagittal plane [21]. It is interesting to know that IVS is curvilinear in shape and not a straight line [22]. The right ventricular (RV) surface of the IVS is gently convex and forms the floor of the RV cavity, whereas the left ventricular (LV) surface is more concave, forming the roof of the LV cavity [22]. Unlike the right ventricle with widely separated inflow and outflow tracts, the LV inflow and outflow tracts are placed one in front of the other (posterior inflow/anterior outflow) [16] (Fig. 2A). This anatomical arrangement splits the LV cavity into a cone-shaped inflow and a tubular outflow tract (Fig. 2B). Inflow into the LV cavity is bounded by the circular fibrous ring of the mitral annulus, whereas the outflow tract is a muscular tunnel formed in large part by the IVS. The concave shape of the IVS on its LV side is crucial for maintaining adequate size and laminar flow through the outflow tract. Hence, incremental septal hypertrophy can significantly reduce the area of the LVOT and is an important substrate of HCM. This effect becomes even more pronounced as the hypertrophy moves closer to the aortic valve creating a bottleneck effect (Fig. 2B). The blood flow through the LVOT is bounded by various important anatomical structures, and they all interact in a complex and synchronous mechanism to provide an unobstructed passage and laminar blood flow (Fig. 3). This implies that aberration in any of these critical structures can lead to LVOT obstruction with profound hemodynamic consequences.

**Fig. 4:**
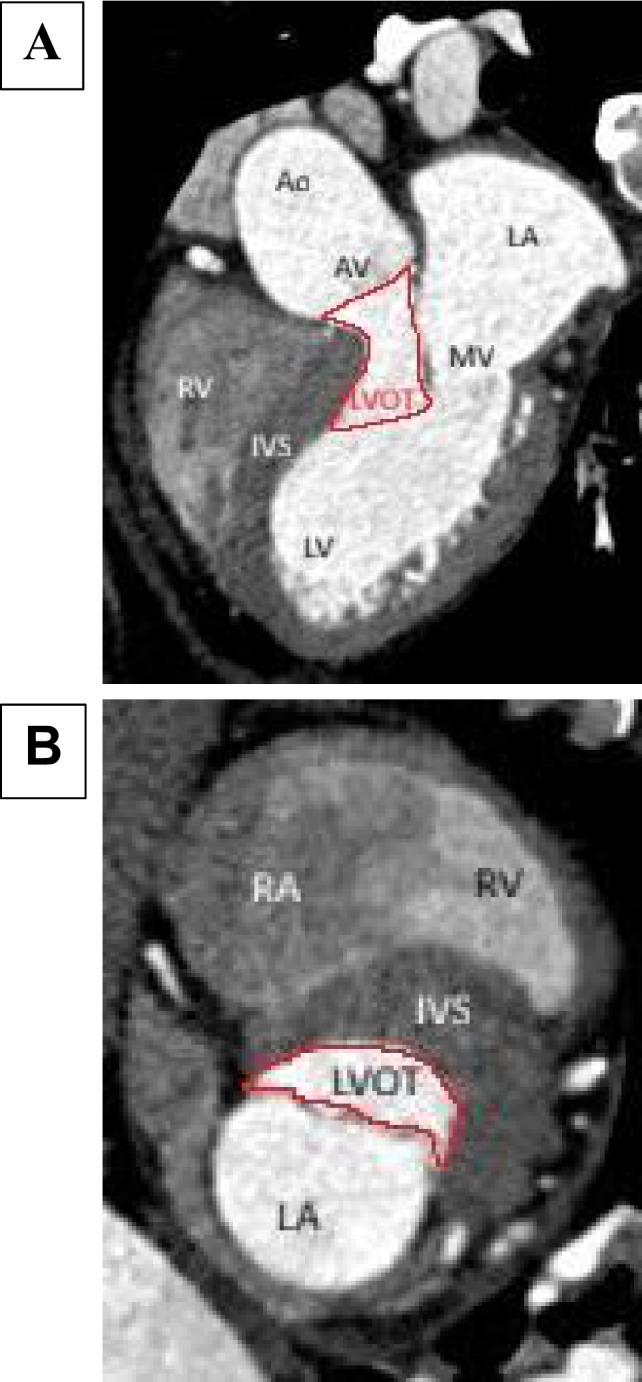
**A** Boundaries of left ventricular outflow tract on a computed tomography image. **B** Elliptical shape of left ventricular outflow tract. Ao-Aorta, AV-Aortic Valve, LVOT-Left Ventricular Outflow Tract, LA-Left Atrium, MV-Mitral Valve, LV-LeftVentricle, RV-Right Ventricle, IVS-Inter Ventricular Septum, RA-Right Atrium

Though the LV ejection begins at the apex, the term outflow tract in strict terms is referred to the anatomical part of the LV just below the aortic valve. It is about 2.5 cm long in healthy adults [21] (Fig. 4A). When viewed end-on, the LVOT is elliptical in shape, and its walls can be imaginarily divided into two parts—anterior two thirds (formed by the muscular IVS) and the posterior one third (formed by the AML) [21] (Fig. 4B). The ends where the anterior and posterior walls meet are marked on either side by the right and left fibrous trigones. The IVS imperceptibly merges with the posterior wall of the LV myocardium without any clear demarcation along an imaginary line from the left fibrous trigone to the apex. This anatomical fact has huge surgical implications as it marks the extent of resection during surgical myectomy.

### Mitral valve abnormalities in HCM

Nearly half of HCM patients have co-existing anomalies of the mitral valve apparatus, and they are more pronounced in patients with genetic inheritance [23]. The anterior leaflet is more commonly involved than the posterior leaflet. Primary chords can be elongated with increased redundancy of the leaflets. There can also be abnormally positioned secondary chords of the AML contributing to SAM and LVOT obstruction [15, 23]. Moreover, various other anomalies can affect the papillary muscles (hypertrophied papillary muscles, anteriorly displaced papillary muscle heads, multiple papillary muscles, and direct attachment to the ventricular surface of the AML). Mitral anomalies combined with septal hypertrophy create perfect conditions for SAM [24].

### Mechanism of SAM

**Fig. 5:**
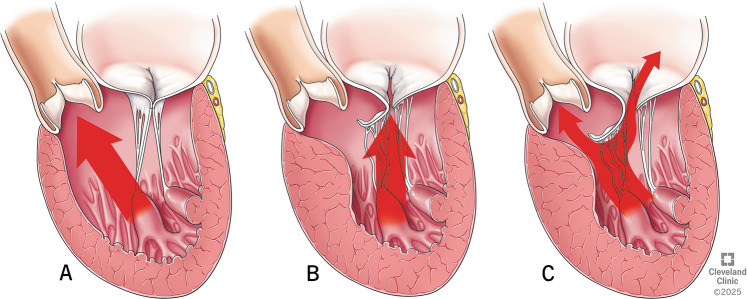
**A** Axis of ejection in a normal heart. **B** Deviated ejection towards mitral annulus in hypertrophic cardiomyopathy. **C** Systolic anterior motion causing left ventricular outflow tract obstruction and mitral regurgitation

In the early stages of systole, papillary muscle contraction causes the tensor apparatus to pull the leaflets posteriorly, clearing the way for ejection towards the anteriorly located LVOT. The axis of ejection in a normal heart is a relatively straight line from apex to the base of the heart (Fig. 5A). In HCM, the hypertrophied septum alters the trajectory of ejection towards the mitral annulus (Fig. 5B). The altered flow vortices hit the mitral valve before it completes its coaptation and sweeps the AML towards the LVOT, leading to SAM and LVOT obstruction [25] (Fig. 5C). Moreover, anomalies of the mitral valvular apparatus, such as abnormal papillary muscle or chordae, pull the AML and the coaptation line more anteriorly. This results in more SAM and its consequences—LVOT obstruction and a posteriorly directed mitral regurgitation (MR) [26].

#### Evolution of mitral procedures for HCM

The evolution of mitral valve procedures in the surgical correction of HCM is an equally interesting journey. While Dr. Morrow strongly believed that adequate septal myectomy would correct mitral insufficiency, Dr. Denton Cooley proposed mitral valve replacement to increase the area of LVOT [27, 28]. Dr. McIntosh from NIH Bethesda did vertical plication of the AML to reduce SAM, but the technique went out of vogue as it led to alteration of the coaptation line and central mitral insufficiency [29]. Dr. Swistel, in his famous RPR operation, did horizontal plication of AML below the aorto-mitral curtain, reducing the length of AML to correct SAM and LVOT obstruction [30]. Anterior mitral leaflet extension (AMLE) is another technique developed by the surgical team from the Netherlands and involves incising the mitral valve and suturing a glutaraldehyde-treated pericardial patch to increase stiffness and width of the AML without lengthening it. The authors published their results with good long-term outcomes [31].

Dr. Messmer stressed trimming of the hypertrophied papillary muscle and Dr. Swistel reported release of abnormal attachments of the papillary muscle to increase its freedom of movements and reduce the anterior drag on the AML [30]. Our team at the Cleveland Clinic devised papillary muscle re-orientation, a technique used to correct bifid or excessively mobile papillary muscle heads that cause LVOT obstruction [32]. Gradually, repair techniques were introduced for anomalies at all levels of the mitral apparatus, and in the current era mitral valve replacement is needed only for patients with intrinsic mitral valve disease not suitable for repair [33].

#### Indications for surgery

**Fig. 6:**
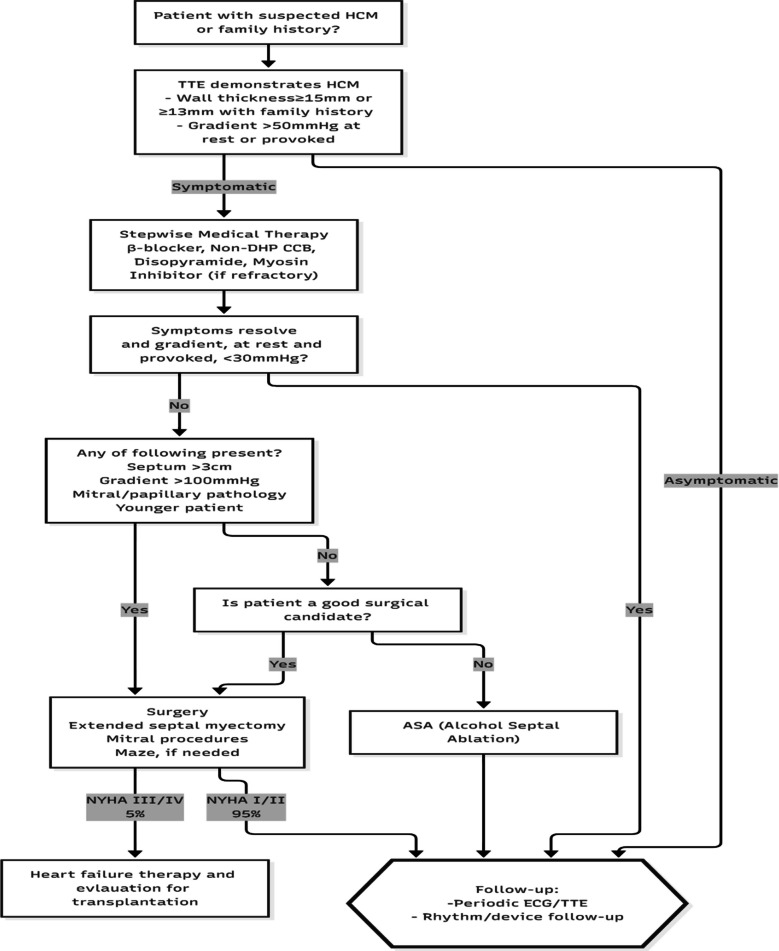
Algorithm for management of hypertrophic cardiomyopathy

HCM is very heterogenous in presentation, and many patients remain asymptomatic and are diagnosed incidentally. These patients can be followed up with electrocardiogram (EKG) and echocardiography at regular intervals or when symptoms develop. Symptomatic patients are started on medical therapy with beta-blockers, calcium channel blockers, and disopyramide [34, 35]. Myosin inhibitors are a new class of drugs which reduce myocardial contractility and improve compliance. They achieve this by reducing the sarcomere force output and are being extensively studied in symptomatic patients not responding to standard medical therapy. Though they have demonstrated benefits in the short term, their safety in terms of left ventricular ejection fraction (LVEF) reduction and long-term outcomes are yet to be determined [36, 37]. Symptom persistence despite maximal medical therapy is an indication for septal reduction therapy. There are two forms of septal reduction therapy—alcohol septal ablation (ASA) and surgical myectomy [38]. Many non-randomized trials have been conducted to compare ASA and septal myectomy. Surgical myectomy has less incidence of right bundle branch block, complete heart block requiring pacemaker implantation, and lesser re-intervention rates [39]. ASA is less effective when the gradient is more than 100 mmHg or when the septal thickness is more than 3 cm [40]. Surgical myectomy has better long-term survival than ASA [41]. Our treatment algorithm for HCM is presented in Fig. 6.

#### Pre-operative planning

Echocardiography plays an important role in pre-operative evaluation, helping to define the type of HCM, identifying associated mitral valve anomalies and the presence of SAM [23]. A clear understanding of the individual septal anatomy and the mechanisms of LVOT obstruction is key to successful surgical outcomes. Though details of echocardiographic assessment of HCM are not the scope of this article, a few echocardiographic details hold important surgical relevance [42]. The two important echocardiographic views are the (1) long-axis view to assess the antero-septum; and (2) the four-chamber view to study the infero-septum [43]. The part of the septum which is hypertrophied in the classical variety of HCM (basal variety) is the anterior septum. It is situated beneath the right ventricular outflow tract (RVOT) and bears the septo-mitral contact lesion. The inferior septum refers to the part of the septum below the membranous septum. The part of the IVS beneath the right coronary cusp is not well imaged using echo and is best assessed by magnetic resonance imaging (MRI).

It is imperative to measure peak gradients both at rest and after provocative maneuvers such as exercise and Valsalva maneuvers, or drugs such as amyl nitrite and dobutamine. The mechanism of SAM and etiologies of MR (SAM-related or intrinsic mitral disease) should also be noted. The degree of MR is assessed with semi-quantitative methods, and the anatomy of MR jets is studied. Associated valvular lesions and the presence of complications like LV apical aneurysm and left atrial appendage (LAA) clot should be looked for in all the patients [23].

#### Conduct of surgery

**Fig. 7:**
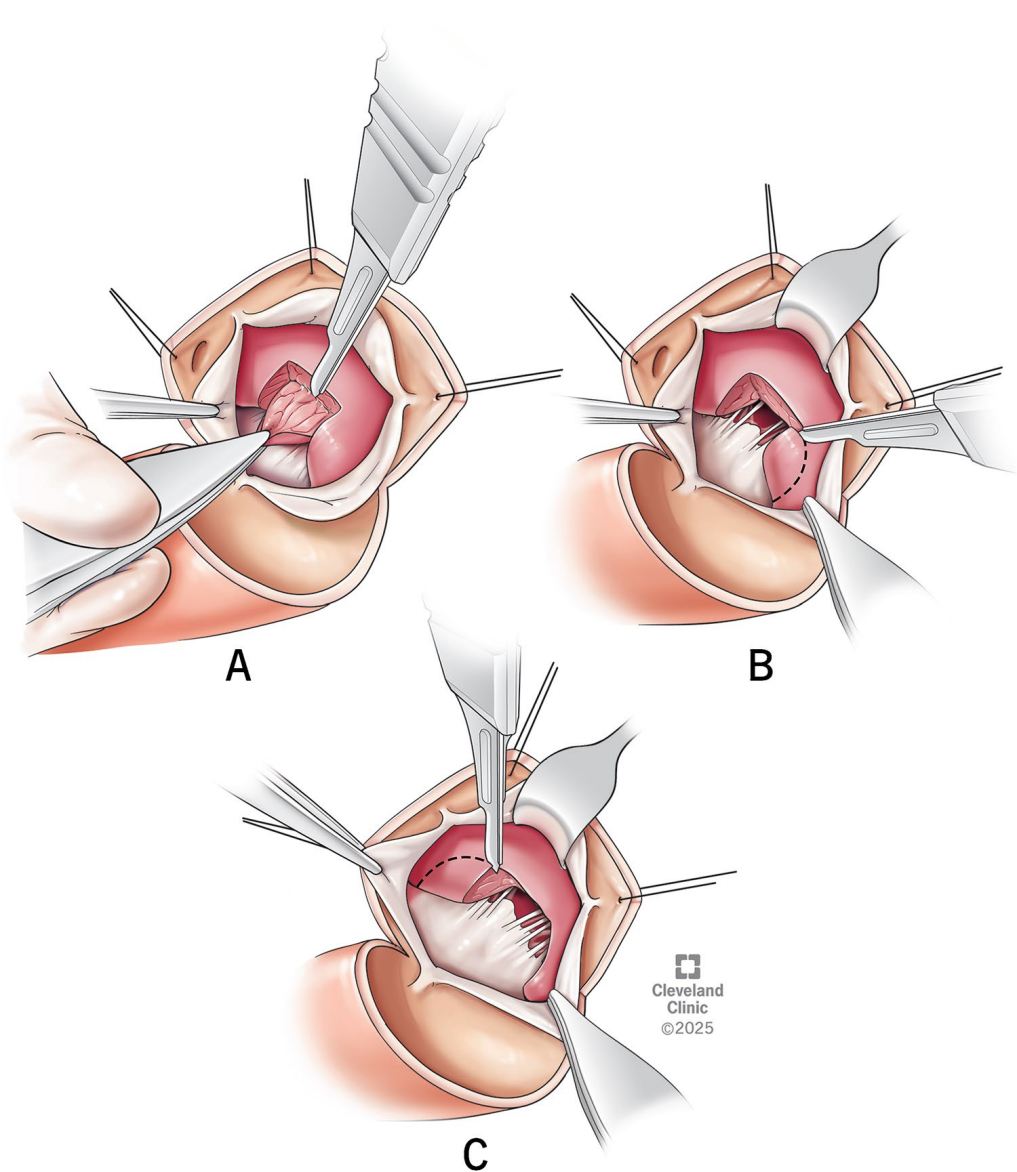
Stepwise demonstration of septal myectomy. **A**-Excision of a rectangle of muscle by two parallel incisions made below the nadir of the right coronary cusp, **B**-Extension of incision towards the right fibrous trigone, **C**-Extension of incision onto the left fibrous trigone

Septal myectomy is a curative therapy for HCM in most of the patients who are symptomatic despite maximal medical management. Median sternotomy is our preferred approach for surgical myectomy. Cardiopulmonary bypass is initiated by ascending aortic and bicaval or right atrial cannulation. After achieving standard cardioplegic arrest, a left atrial vent is placed via the right superior pulmonary vein. Transverse aortotomy is made and a thin malleable retractor is used to retract the aortic valve and help in exposing the ventricular cavity. Intracardiac ultrasound of the septum (Aplio i900—Toshiba Medical) can be done to assess the thickness of the septum and to guide the extent of resection. The septum, AML, and sub-valvular apparatus are systematically examined to look for any abnormalities in the mitral apparatus contributing to LVOT obstruction. Myectomy is performed in a three-step approach. As a first step, two parallel incisions are made below the nadir of the right coronary cusp, which are 10 mm apart, 5 mm deep, and 15–20 mm long towards the apex. These incisions are connected using a #15 blade, and a rectangle of muscle is excised (Fig. 7A).

In the second step, the incision is carried to the right towards the right fibrous trigone, staying well below the membranous septum. It is important to recall the thickness of the inferior septum measured on the pre-operative echocardiogram, as the inferior septum is generally thinner than the anterior septum (Fig. 7B). The third step consists of extending the anterior edge of the first incision onto the left fibrous trigone to excise another rectangle of muscle (Fig. 7C). The incision here can be carried close to the left coronary cusp of the aortic valve, as there is no risk of conduction tissue injury. Care should be taken to leave a thin ridge of fibrous tissue for the malleable retractor to hold the LV wall, and it can be excised at the end after completing the deeper resections. As each rectangular block of the septal muscle is excised, they are arranged on a towel in the same orientation to re-create the septum as it has been removed. This process is repeated deeper into the ventricle at the level of the mid-papillary muscle. The extent of resection is determined by the location of the septal hypertrophy seen on trans-esophageal echocardiogram. Resection near the base of the heart is in the shape of an arch between the left and right trigones. As we go deeper, the margins of the papillary muscle serve as a guide for resection. The distal extent of resection varies in individual patients depending upon the degree and depth of hypertrophy. After the resection is complete, we weigh the mass of myocardium removed, and the ventricular cavity is irrigated with saline to remove any debris from the resection. The trans-aortic approach is our preferred approach for all varieties of HCM. When the exposure is inadequate, additional maneuvers can be added, such as opening the left pleura, placing stay sutures on the inferior aspect of the aortotomy, or using a sponge on a stick to push the ventricle gently from above in a clockwise direction to bring the apex into view. For deeper resections in the apex, we use long knife handles and thoracoscopic instruments to improve the reach of the surgeon [18, 23].

#### Mitral valve procedures

**Fig. 8:**
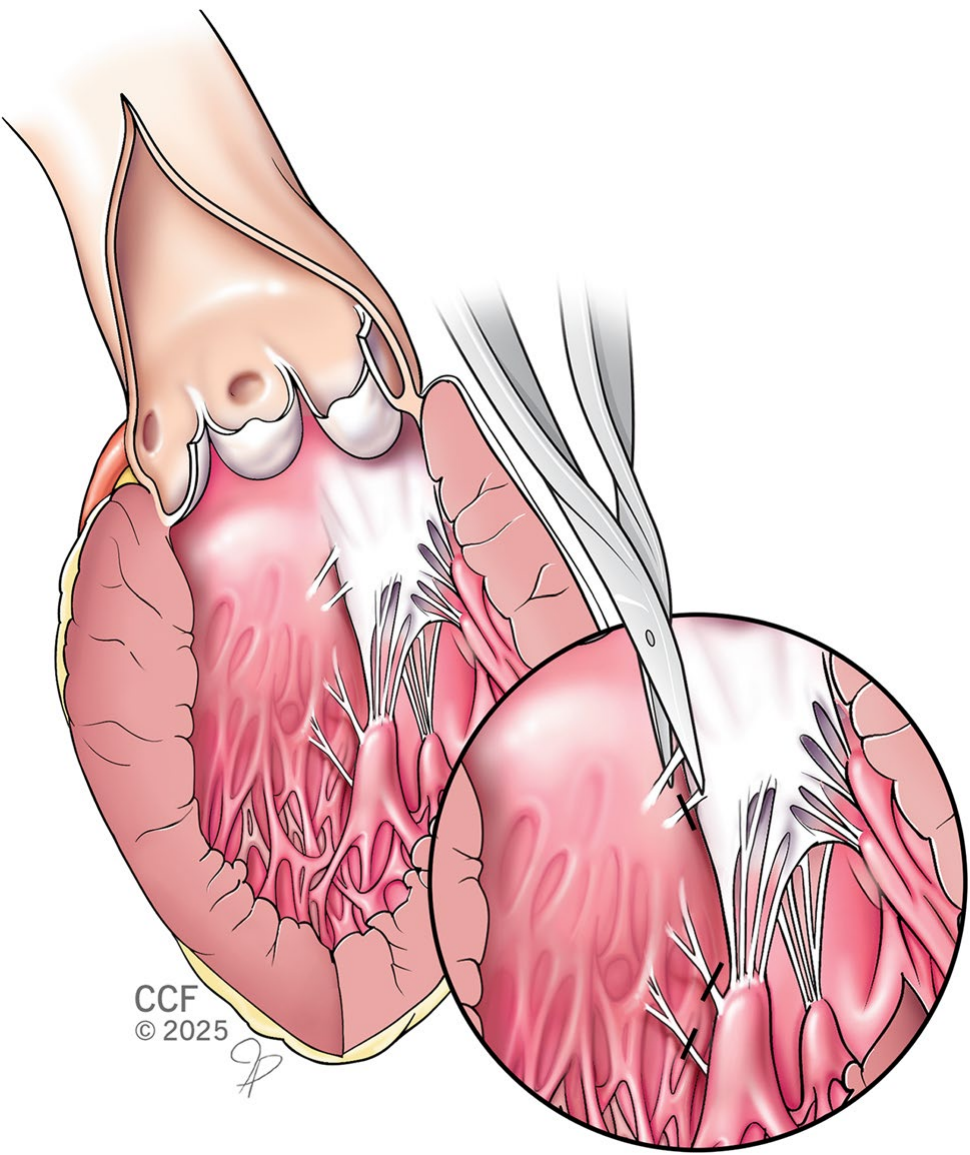
Division of abnormal secondary chords

**Fig. 9:**
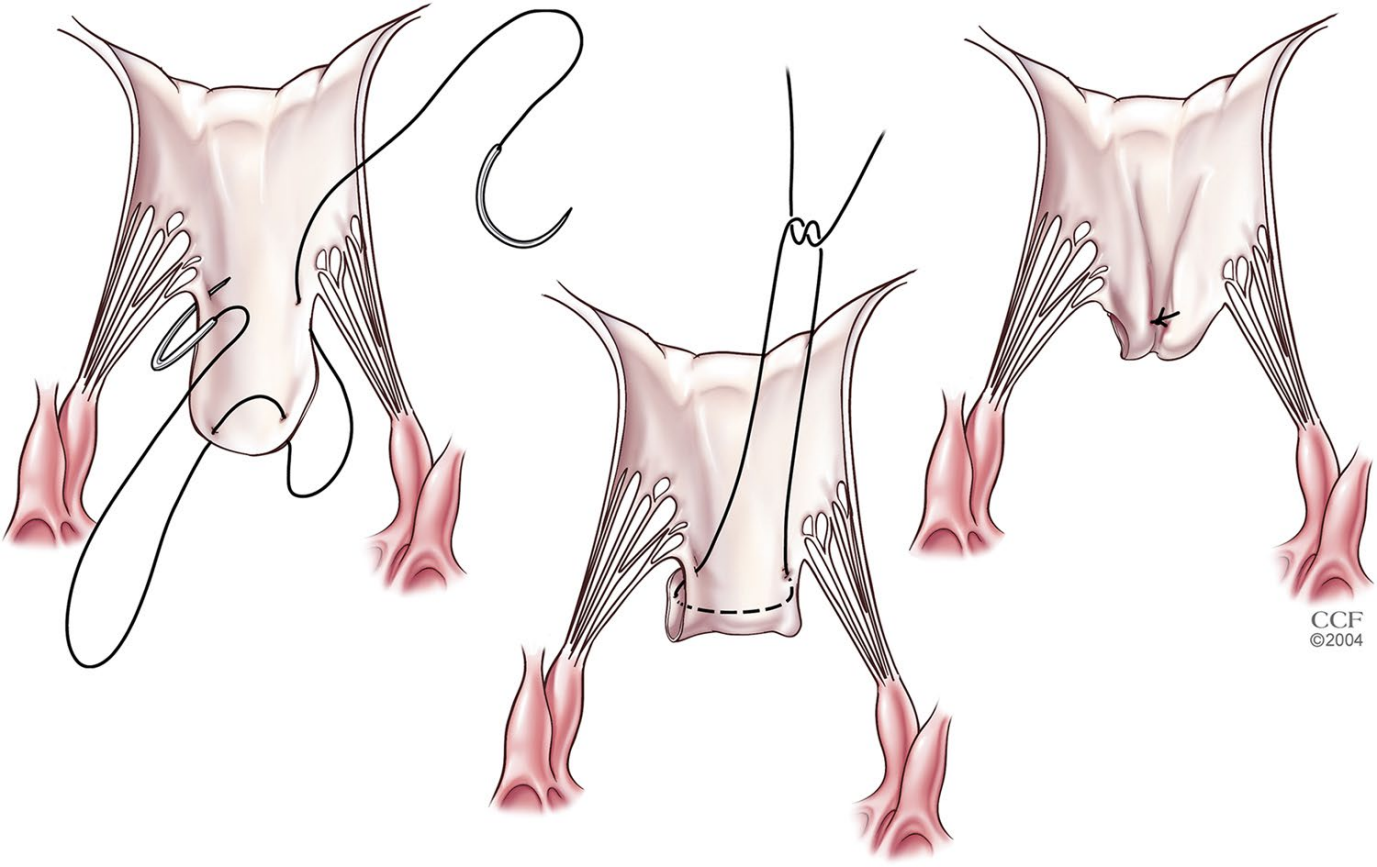
Folding valvuloplasty of anterior mitral leaflet

Any associated mitral valve abnormalities should be assessed intra-operatively and treated. Abnormal chords attached to the septal wall, or the AML, should be severed. Abnormally thickened secondary chords pulling the AML into an anterior position should be identified and resected (Fig. 8). Elongated anterior leaflets can be treated with folding valvuloplasty or free edge resection (Fig. 9).

#### Papillary muscle relocation and partial resection

**Fig. 10:**
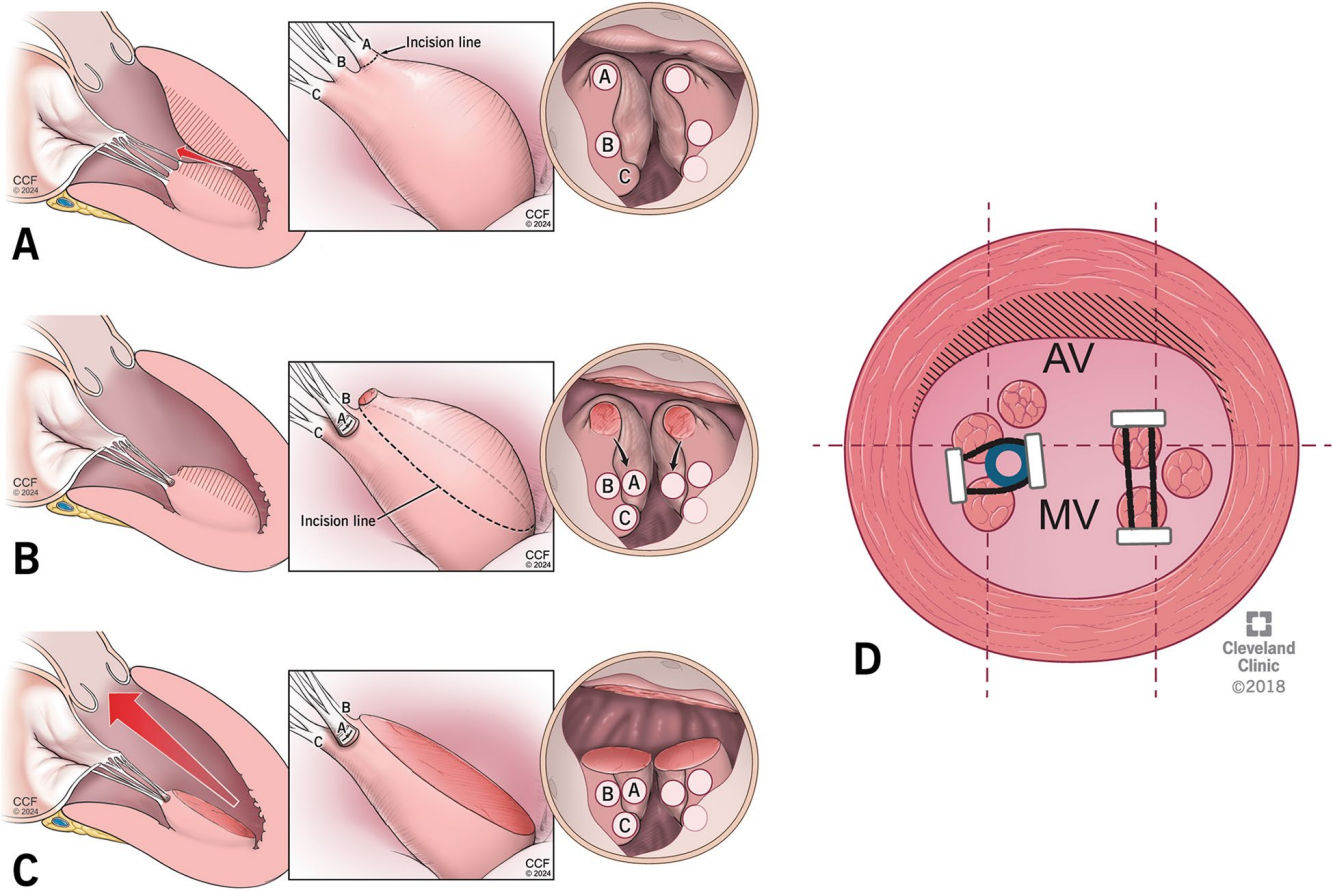
Papillary muscle head relocation and partial resection. AV-Aortic Valve, MV-Mitral Valve, **A**, **B**, **C**- Papillary Muscle Head

Hypertrophied, bulky, and anteriorly displaced papillary muscle can cause LVOT obstruction. They need to be corrected to relieve LVOT obstruction. Bulky papillary muscles projecting into the LVOT are partially resected. Any muscle head with chordal attachment from the resected part is relocated to the posterior heads of the same papillary muscle. The aim of this intervention is to restore the plane of the papillary muscle to a hemodynamically favorable posterior position away from the LVOT [44] (Fig. 10).

#### Papillary muscle re-orientation

**Fig. 11:**
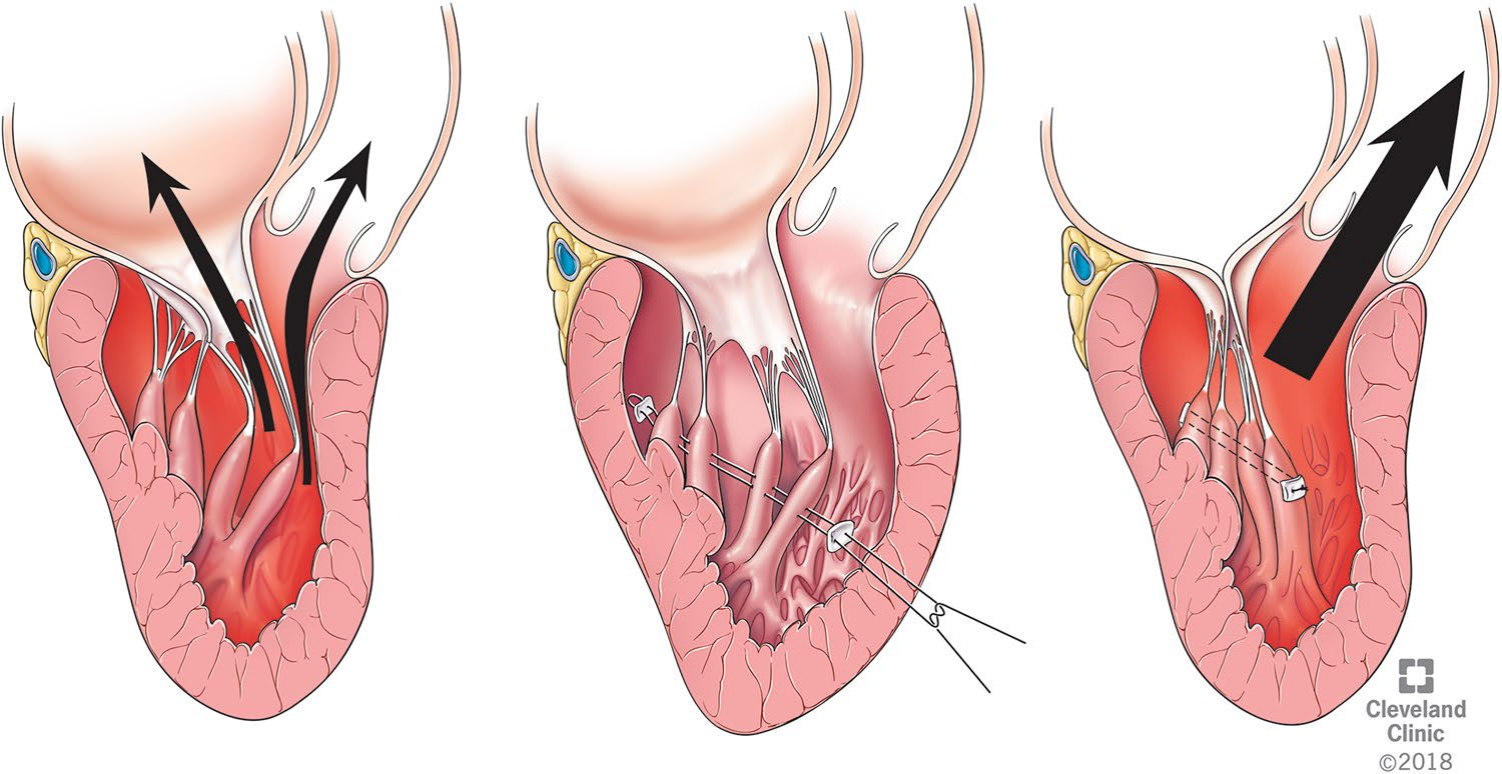
Papillary muscle re-orientation

Muscle heads of anterior and posterior papillary muscles can be abnormally displaced more anteriorly, leading to LVOT obstruction with or without septal hypertrophy. Muscle heads from the posterior papillary muscle are more commonly involved than the anterior papillary muscle. Papillary muscle re-orientation (Fig. 11) involves suturing the excessively mobile muscle heads to less mobile posterior heads of the same papillary muscle, thereby pulling the anterior heads away from the LVOT. Care must be taken to avoid twisting of the muscle heads, trapping of chords, and ischemia while suturing. Papillary muscles with a wider base of attachment to the posterior wall are necessary for successful re-orientation [32].

### Assessing the adequacy of repair

After separating the patient from cardiopulmonary bypass (CPB), intra-operative transesophageal echocardiogram (TEE) is performed to assess the adequacy of resection and correction of SAM/MR. Assessment is done both in resting state and after pharmacological provocation using isoproterenol infusion to achieve tachycardia and hypotension mimicking a state of dynamic obstruction. We accept a provoked peak gradient of less than 30 mmHg and less than 2+ MR. If these endpoints are not met, the patient will be placed back on CPB and residual lesions are corrected [18, 23]. We use Doppler to measure gradients while other centers use needle measurements across the septum to measure gradients, and both have given equal results [23, 45].

### Mid-ventricular and apical sub-types

Apical and mid-ventricular varieties are characterized by the absence of basal septal hypertrophy and the presence of apical and mid-ventricular hypertrophy [46]. These patients suffer from a very small ventricular cavity with poor compliance, reduction of end diastolic volume, and eventually reduced cardiac output. Dr. Schaff et al. from Mayo Clinic have developed the trans-apical surgical myectomy, wherein a 7-cm incision is made lateral to the left anterior descending (LAD) coronary artery at the apex and muscle resection is done trans-apically to increase the size of the LV cavity [47, 48]. The trans-apical approach is a good alternative and a necessary tool in the armamentarium of a myectomy surgeon. It is used in the following situations—apical HCM, as a combined approach in mid-ventricular HCM with suboptimal trans-aortic exposure, HCM with a small aortic root, and in re-operative cases with inadequate exposure [48, 49]. We use the trans-aortic approach as the primary approach for all varieties of HCM, and the trans-apical approach is reserved for apical HCM where exposure is inadequate via the trans-aortic route [50].

## Results

Surgical myectomy remains the time-honored treatment for HCM patients with drug-refractory limiting symptoms due to LVOT obstruction. Based on > 50 years’ experience, surgery reliably reverses disabling symptoms by permanently abolishing impedance to outflow, leading to normalization of LV pressures and preserved systolic function.

A consortium of ten multi-disciplinary high volume HCM centers in six countries reported their experience of about 11,000 patients over the last 15 years [51]. The perioperative mortality (operative 30-day mortality) was 0.6%, making surgical myectomy one of the safest open-heart procedures in the present day. In this large cohort, the average age at surgery was 54 years; men were predominant than women (55:45). Pacemaker implantation for heart block was 4%, iatrogenic ventricular septal defect (VSD) was 0.3%, and mitral valve replacement was 1.8%. Extended myectomy relieved symptoms in >90% of patients by ≥ 1 New York Heart Association (NYHA) functional class, returning most to normal daily activity and with a long-term survival benefit [51]. When performed in high-volume dedicated HCM centers, the in-hospital operative mortality for myectomy (0.6%) is 12-fold less compared to low-volume centers (6–15%) [51].

Over the last three decades, our team at the Cleveland Clinic has performed more than 4000 myectomies until now. We conducted a detailed analysis of our patients between 2005 and 2015 [43]. Within this study period, 1549 patients underwent surgery for HCM. Their mean pre-operative peak LVOT gradient was 63 ± 4.6 mmHg, and it reduced to 15 ± 8.9 mmHg after surgery. Mean aortic cross-clamp time was 28 ± 10 min, and mean mass of muscle resected was 8.1 ± 3.7 g. Complications include the need for pacemaker insertion in 4.2% of the patients, iatrogenic VSD in two patients, and an overall operative mortality of 0.38%. Mean hospital stay was 6 days, and the majority of the patients are in functional class I in their post-operative follow-up.

### Pediatric HCM

HCM is one of the most common causes of sudden cardiac death in the young. The presentation in the pediatric population encompasses a broad spectrum, from asymptomatic patients to those with limiting heart failure symptoms and even sudden cardiac death [52, 53]. Symptomatic HCM in kids is known to have a more malignant course than adults, with early death rates and complications [53, 54]. Pediatric surgical myectomy presents unique challenges because of the smaller aortic opening and ventricular cavity leading to poor exposure. Thus, there is a higher risk of residual lesions, and complications such as VSD and complete heart block (CHB) are more common [33]. Over a period of 16 years, we operated on 398 patients, less than 18 years of age for HCM with no operative mortality. During the follow-up of 15 years, there were nine (2%) deaths, 29 (7%) patients had redo myectomy, and eight (2%) required heart transplantation [55]. The Mayo Clinic reported similar results—their overall survival and freedom from re-operation at 20 years after successful septal myectomy were 92.4% and 72.7%, respectively [56]. Long-term results in the pediatric age group are improving. Early surgical referral and lower thresholds for ICD usage are important considerations, given the early progression and higher incidence of sudden cardiac death in this group of patients [57]. 

## Conclusion

Septal myectomy is a curative therapy for most patients of HCM who are symptomatic despite maximal medical management. Clear understanding of individual septal anatomy and mechanisms of LVOT obstruction is key to successful surgical outcomes. Septal myectomy can be performed safely with excellent long-term results.

## Data Availability

All data is in public domain.
